# Performance on verbal fluency in late-onset schizophrenia is more preserved than in early-onset schizophrenia

**DOI:** 10.1192/j.eurpsy.2021.1409

**Published:** 2021-08-13

**Authors:** E. Abdullina, G. Rupchev, M. Savina, Y. Panikratova, E. Kucherova, V. Sheshenin, M. Morozova

**Affiliations:** 1 Department Of Gerontological Psychiatry, FSBSI Mental Health Research Center, Moscow, Russian Federation; 2 Laboratory Of Psychopharmacology, FSBSI Mental Health Research Center, Moscow, Russian Federation; 3 Laboratory Of Neuroimaging And Multimodal Analysis, FSBSI Mental Health Research Center, Moscow, Russian Federation

**Keywords:** verbal fluency, late-onset schizophrenia, cognition

## Abstract

**Introduction:**

According to the literature, cognition may be more preserved in late-onset schizophrenia (LOS) compared to early-onset schizophrenia (EOS), but data are limited.

**Objectives:**

To compare performance on cognitive tests in LOS and EOS.

**Methods:**

LOS patients (n=14, mean age 58.1±8.2, 13 females, illness duration 1.07±1.5 years) and age-comparable controls (n=17, mean age 55.3±7.8, 12 females), EOS patients (n=25, mean age 20.7±3.9, 25 males, illness duration 0.75±0.62 years) and age-comparable controls (n=15, mean age 22.9±2.3, 15 males) underwent the Brief Assessment of Cognition in Schizophrenia (BACS) comprised of six subtests: Verbal Memory, Digit Sequencing, Verbal Fluency, Token Motor Task, Symbol Coding, and Tower of London. The Mann-Whitney U test with Bonferroni correction for multiple comparisons was applied (p <.05/8, i.e. p <.006).

**Results:**

Compared to LOS, EOS patients had lower score on Verbal Fluency (VF): U=78, p=.004; mean T-scores are 43.5±9.5 and 33.6±12.6 for LOS and EOS, respectively. Additionally, we compared VF performance in each clinical group with age-comparable controls and revealed significantly lower performance in both LOS (U=37.5, p=.001) and EOS (U=56.5, p=.000).

**Conclusions:**

Performance on VF is deteriorated in clinical groups, but may be more intact in LOS compared to EOS. This result is of particular interest because low performance on VF is considered as a cognitive endophenotype of schizophrenia. Performance on VF requires preserved executive functions, language, and processing speed. Our results are in line with the idea that LOS and EOS may be different subtypes of schizophrenia. Limitation of this study is that the clinical groups are not sex-matched.

